# Reply

**DOI:** 10.1097/QAI.0000000000002127

**Published:** 2019-07-03

**Authors:** Yi Zhang, Till Bärnighausen, Nir Eyal

**Affiliations:** a Stanford University School of Medicine, Stanford, CA; b Heidelberg Institute of Global Health, University of Heidelberg, Heidelberg, Germany; c Africa Health Research Institute, Durban, South Africa; d Department of Global Health and Population, Harvard T. H. Chan School of Public Health, Boston, MA; e Center for Population-Level Bioethics, Rutgers University, New Brunswick, NJ

***To the Editors***

The respondents claim that we uphold the “failed approach of rationing treatment”. However, rationing—the allocation of scarce resources—is an unescapable fact of life. When resources for HIV antiretroviral treatment (ART) are so abundant that everyone who is HIV-positive—now and in the future—can receive immediate ART, immediate ART for all is indeed the best possible option—as we explain clearly in our article.

But when resources for ART are scarcer than this maximum, to ration them explicitly and ethically is both rational and fair.^1,2^ Scarcity of resources for ART is not a necessary future, but it is unfortunately becoming increasingly likely, as donor commitments for ART are shrinking.^3–6^ We can and should jointly fight this development. But if we do not succeed and the resources for immediate ART for all are simply not available, there will be no alternative to rationing. The only question is whether to ration implicitly—that is, make everyone eligible for ART upon diagnosis and let coincidence or, worse, power and privilege dictate who actually receives this life-saving resource—or explicitly, by a certain fair criterion.

We proposed that the fairest criterion would allocate this resource to the sickest patients. A simple thought experiment can illustrate our reasons for this recommendation: In a world with two people who are HIV-positive, one has recently become infected and is still healthy and the other one has been living with HIV for a long time and is sick. The HIV treatment guidelines state that both patients should receive ART. But if we only have resources to provide ART to one patient—that is, “ART budgets cannot cover all patients”^7^—who among the two should we prioritize?

As we explain in our article, in this “competition between immediate eligibility and later eligibility for ART, later eligibility is morally superior, by the lights of all leading rival ethical approaches bearing on this question,”^7^ that is, the sickest patient should receive ART first. Prioritizing the sickest tends to save the most lives and life years per dollar. The sickest patients are also probably the most desperate and, frequently, the most disadvantaged because there is often a structural reason why someone was unable to receive ART early on in their disease progression. Finally, the sickest patients are also the most infectious. Our respondents write that “money spent on immediate ART eligibility decreases the number of people who reach those later stages by stopping the spread of HIV to new individuals”. However, as we write in our article, “while immediate ART is highly efficacious against onward transmission of HIV, so is late ART. Indeed, with the exception of initial weeks of primary infection (during which patients rarely reach clinics), viral load and infectiousness increase steadily as HIV progresses.”^7^ Thus, if “ART budgets cannot cover all patients,”^7^ prioritizing the sickest in the late stages of HIV disease implises the largest transmission reductions.

The combined result of these different facts about ART is that prioritizing the sickest is preferred by some ethical theories and opposed by none. Far from it being the case that our “conclusion is not supported … by basic ethical principles”, a wide range of basic ethical theories, cited in our article, would prioritize a sick patient over a healthy one. As we stated in our article, “[w]e wholeheartedly endorse providing ART to all patients where possible.”^7^ When, however, this highly desirable goal cannot be reached, prioritizing the sickest patients is morally superior to other rationing schemes in this setting.
